# 3D Cell Cultures as Prospective Models to Study Extracellular Vesicles in Cancer

**DOI:** 10.3390/cancers13020307

**Published:** 2021-01-15

**Authors:** Guillermo Bordanaba-Florit, Iratxe Madarieta, Beatriz Olalde, Juan M. Falcón-Pérez, Félix Royo

**Affiliations:** 1 Center for Cooperative Research in Biosciences (CIC bioGUNE), Exosomes Laboratory, Basque Research and Technology Alliance (BRTA), E48160 Derio, Spain; gbordanaba@cicbiogune.es (G.B.-F.); jfalcon@cicbiogune.es (J.M.F.-P.); 2 TECNALIA Basque Research and Technology Alliance (BRTA), E20009 Donostia San Sebastian, Spain; iratxe.madarieta@tecnalia.com (I.M.); beatriz.olalde@tecnalia.com (B.O.); 3 Centro de Investigación Biomédica en Red de Enfermedades Hepáticas y Digestivas (Ciberehd), E28029 Madrid, Spain; 4 Ikerbasque, Basque Foundation for Science, E48009 Bilbao, Spain

**Keywords:** 3D culture, extracellular vesicles, tumoral cells, cancer, therapy

## Abstract

**Simple Summary:**

3D cell cultures are a qualitative improvement in cancer research because these models preserve cancer physiological characteristics better than traditional bi-dimensional cultures. Moreover, they facilitate the study of complex 3D interactions using extracellular matrices and the co-culture of different cell types. In this manner, the cells can contact themselves in a fully physiological but also controlled arrangement. In the context of tumor interactions, extracellular vesicles are essential in number of key aspects in oncology: as major interactors with extracellular matrix, as cell-to-cell messengers, as carriers of diagnostic-valuable biomarkers, and as target-specific treatment-deliver agents. The present article aims to discuss the findings achieved using 3D culture models in oncology. We further review the involvement of extracellular vesicles in the pathogenesis of cancer as well as their potential use in diagnostics and therapeutics.

**Abstract:**

The improvement of culturing techniques to model the environment and physiological conditions surrounding tumors has also been applied to the study of extracellular vesicles (EVs) in cancer research. EVs role is not only limited to cell-to-cell communication in tumor physiology, they are also a promising source of biomarkers, and a tool to deliver drugs and induce antitumoral activity. In the present review, we have addressed the improvements achieved by using 3D culture models to evaluate the role of EVs in tumor progression and the potential applications of EVs in diagnostics and therapeutics. The most employed assays are gel-based spheroids, often utilized to examine the cell invasion rate and angiogenesis markers upon EVs treatment. To study EVs as drug carriers, a more complex multicellular cultures and organoids from cancer stem cell populations have been developed. Such strategies provide a closer response to in vivo physiology observed responses. They are also the best models to understand the complex interactions between different populations of cells and the extracellular matrix, in which tumor-derived EVs modify epithelial or mesenchymal cells to become protumor agents. Finally, the growth of cells in 3D bioreactor-like systems is appointed as the best approach to industrial EVs production, a necessary step toward clinical translation of EVs-based therapy.

## 1. Introduction

In recent years, the number of scientific groups dedicated to the study of extracellular vesicles has grown notably, and with it the amount of published information describing extracellular vesicles (EVs) physiology. Released by all types of cells, they are an important tool to study cell’s biology, and to look for biomarkers. Cancer research is one of the main fields that can benefit of the study of EVs associated to tumors. In fact, the vesicle-mediated cell-to-cell crosstalk seems to be important in every step of cancer progression [1]. In parallel, the study of cancer biology had evolved itself along the last years towards culture models that reflect the biological complexity of tumoral cells and their interactions with the extracellular matrix. The reason is that the traditional bidimensional (2D) cultures differ from tridimensional (3D) cultures in their morphological characteristics, proliferation rate and degree of differentiation, the level of cell-to-cell interaction and cell-to-matrix, as well as their resistance to drugs [2,3]. However, the application of complex culture models to unravel the role of EVs in cancer research has not been yet popularized among EVs research, given the difficulties that this type of cultures presents, both technically and in terms of cost. Nevertheless, several studies have highlighted the importance of 3D cultures in the study of EVs in cancer research [4,5,6]. In this article, we aim to emphasize the contribution of those studies as a fundamental path to understand the involvement of EVs in cancer physiology and to pinpoint possible applications to the clinical oncology. To help to understand the background of this review, we are providing a short introduction to the different roles that EVs play in cancer and cancer therapy, and a brief description of the different 3D cultures employed to study tumoral cells. Afterwards, the review summarizes different studies that employ 3D culture systems to elucidate the role of EVs in cancer biology, diagnosis and therapy.

## 2. The 3D Cultures as a Physiological Model of Tumoral Cells

For many years, in vitro models were based on 2D monolayers of immortalized human cancer-derived cell lines. The popularization of 3D culturing has come with the observation that this type of cell cultures often retain heterogeneity. This feature allows the study of tumor evolution. Moreover, 3D cultures offer advantages over conventional monolayered cell cultures including preservation of the topology and cell-to-matrix interactions [7,8]. On the other hand, the application of 3D cultures is also challenging, given the difficulties to stabilize the cultures, and the requirement of specific material to perform the culture. In Table 1, we present a comparative between 2D and 3D cultures characteristics. In spite of the difficulties, 3D cultures become a great model to study the interplay between cancer and non-cancer cells in order to unveil biological mechanisms involved in cancers initiation and progression [9]. Spheroids are probably the type of 3D culture most commonly used. Spheroid formation methodologies can be divided into two categories: scaffold-based models, either incorporating materials which are components of the matrix (collagen, fibronectin, agarose, laminin, and gelatin) [10], or synthetic materials that provide cell support [11], and scaffold-free models that comprise non-adherent and in suspension cells, which are forced to aggregate and form spheroids [12].

One of the first applications of 3D cultures was the study of tumorigenesis. Typically, the cells are cultured in a mouse sarcoma-derived gel (i.e., Matrigel^®^). Other alternatives exist, such human leiomyoma discs and their matrix (Myogel). This has been commercialized for in vitro assays such IncuCyte^®^, spheroid and sandwich assays [15].

3D culture models grown in vitro from cancer stem directly or from primary tissues are a more evolved form of organoids [16]. The latter option has an attractive potential for personalized medicine. For instance, when comparing organoids derived from primary colorectal tumors and metastatic lesions isolated from the same patients, it has been observed that they share common mutations. This implies that the driver alterations preceded metastatic dissemination [17]. Organoids display greater number of features and functions of their original organs, such architecture and gene expression, reason why they have a prospective potential for the cancer research. The combination of organoids with the co-culture of multiple cells can mimic the tumor immune microenvironment, including key features like immune check point [18]. Organoids derived from different mouse or human tumors have now been widely adopted to investigate different types of cancer, for example. colorectal cancer [19]. Moreover, by culturing organoids in the proper media conditions, they could serve as a model of the three most common subtypes of liver cancer: hepatocellular carcinoma, cholangiocarcinoma, and combined hepatocellular cholangiocarcinoma [20]. Several other models such as prostate, brain or kidney organoids have been stablished and largely reviewed in [21].

In addition to organoids, other kinds of 3D cultures have been developed. 3D bioprinting can be defined as a layer-by-layer deposition of biomaterial, such as tissue spheroids, cell pellets, microcarriers, decellularized extracellular matrix, and cell-laden hydrogels, in a well-defined structure to generate viable 3D cultures. In the last decade, the bioprinting technologies have undergone remarkable advancements [22]. Current trends utilizing scaffold technologies aim at capturing more of the micro-environmental cues than other model systems [23,24]. The scaffolds may act as a surrogate for the missing ECM, representing the available space of tumor tissue, providing the physical support for cell growth, adhesion, and proliferation, and causing the cells to form an appropriate spatial distribution and cell-cell or cell-ECM interaction [2].

A wide range of techniques are used in generating different scaffolds, including solvent casting/particulate leaching, freeze-drying, phase inversion, electrospinning, stereolithography, selective laser sintering, shape deposition manufacturing, 3D printing, robotic microassembly, and fused deposition modeling [25]. Among these techniques, freeze drying, phase inversion, and fiber electrospinning are used most of the times. Commonly materials used for tumor cells 3D culture are a laminin-rich basement membrane extract gelatin (for instance Matrigel, Myogel or Cultrex BME) [26], silk fibroin proteins [27], hyaluronic acid [28], collagen [29], or decellularized material [30,31]. Greater understandings of tumor microenvironment have identified ECM to play critical roles in orchestrating drug resistance, disease progression and tumor metastasis [32]. Two independent articles have revealed that lack of ECM during 3D culture could not elicit pathological phenotypes observed in vivo, such as maintaining cancer-associated fibroblasts behaviors [33] or stromal barriers [34]. Scaffold based 3D cell culture, using a biological basement membrane, captures many aspects of the spatial cues (cell-to-cell communication, cell-to-matrix adhesion, and physical characteristics) and provides a unique compromise between complexity and practicality [35]. The choice of a biological scaffold is not simply to deliver an anchorage site for cells but also to provide a complex structure enabling communication linked to cell behavior and function [36]. The formation of 3D structures within the culture also reproduces aspects of the nutrient and oxygen gradients found across in vivo tumors. It should be considered that those 3D scaffolds can be used not only to simulate the microenvironment but alto to assess drug research. Recently publications have showed the ability of decellularized ECM materials to encapsulate and controlled delivery of different drugs such as dexamethasone [37] or doxorubicin [38]. So, 3D scaffold can have drug-carrier functions in therapeutic applications related to testing drugs and in predicting treatment efficacies.

**Figure 1:**
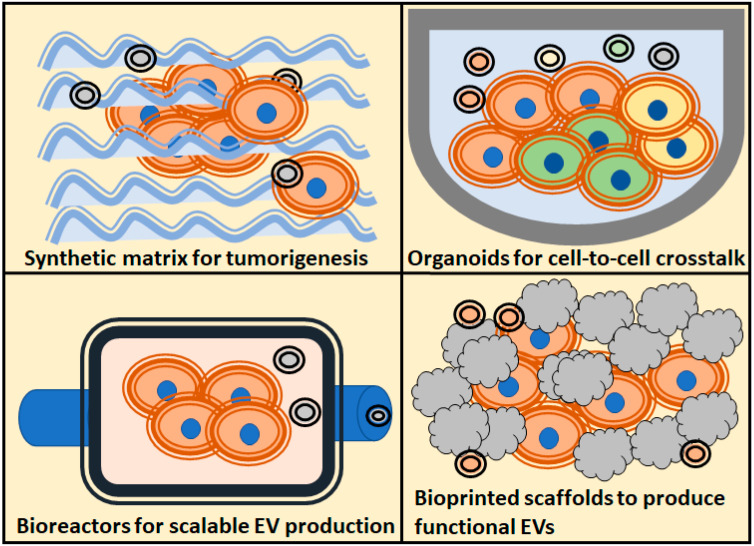
Schematic overview of the most popularized 3D culture techniques, and the main assays regarding extracellular vesicles (EVs) applications to the study of tumors biology, use of EVs as therapeutic agents, study of tumorigenesis and cell-to-cell crosstalk.

In this review, we will find examples of the different 3D strategies employed to study the different roles of extracellular vesicles in cancer, and which models are the most employed to solve each question regarding the role of EVs in tumorigenicity processes (Figure 1).

## 3. Extracellular Vesicles in Cancer Research

Since the first descriptions of EVs and their different types, it has been reported that tumoral cells secrete vesicles. These vesicles participate in the cellular cross-talk with the cellular matrix [39] and cancer cells are rather effective in vesicular-mediated intercellular transfer [40]. Actually, this transfer is a requirement of tumoral cells to stablish a connection with the surrounding matrix and to actively regulate processes involved in cancer progression and autocrine/paracrine oncogenesis. Indeed, EVs play an important role in reprogramming stromal cells, modulating the immune system, and promoting angiogenesis (reviewed in [41]). Moreover, the dependency of tumors on vesicular communication also concerns the preparation of an extracellular niche for metastasis [42,43,44]. For more detailed reviews about the implications of EVs in tumor biology and progression there are very interesting reviews published along the last years [5,45,46], and there are also specific publications related to prostate cancer EVs [6,8].

Tumor communication with targeted cells has a tight reliance on EVs. For this reason, many opportunities for diagnostic and treatment appeared with the analysis and the manipulation of EVs. It is well-known that most malignancies are associated with an increase of circulating EVs. Moreover, it has been described that in different tumor models exist a correlation between tumor volumes and the concentration of circulating EVs in blood [47]. These EVs carry a cargo with precious information about the tumor, and they have become the substrate for biomarker digging in all types of malignancies [48,49,50] including prostate cancer [51,52,53].

In parallel, many studies have focused on how circulating EVs are being captured by tumoral cells, and how to increase the specificity of the capture of EVs by tumoral cells using different strategies. An interesting line of research studies EV membrane decoration with proteins. For example, LAMP2b protein has been successfully fused to ligands specific for brain, angiogenic endothelium, or IL3 receptors on myeloid leukemia cells to direct EVs specifically toward the selected tissue [54,55,56]. Other considerations, such as biodistribution, permeability of tumoral cells, and ability to deliver the cargo shall be taken in account and have been largely reviewed in [57].

All these examples unveil the need to deep into the understanding of the three key aspects of cancer research: pathogenesis, diagnosis, and therapy. Below, we will be described how 3D models have contributed to gain knowledge in those topics, and what are the most interesting results obtained so far. For clarity, we talk about EVs even if the original work refers to as exosomes; in most of those articles, the isolation methods employed actually enriched the preparation in small vesicles, but not necessary in vesicles originated from the endocytic pathway.

## 4. Production of EVs in 3D Cultures

As we mentioned previously, 3D culturing allows cells to grow in a physiological topology, and organoids and spheroids still release EVs. More importantly, the EVs produced are functional; EVs released by pancreatic cancer organoids can activate p38 MAPK and induce the expression of F-box protein 32 and UBR2 in myotubes [58]. When compared to 2D conformations, 3D cultures show an increase in EVs release in the case of colorectal cancer stem cells [59]. For colon cancer organoids, the presence of APC mutations that activate WNT pathway enhanced the EVs release in Matrigel-based cultures. This release was probably also favored by the presence of collagen (a component of the extracellular matrix), since it is part of this type of gel [60]. Moreover, another plausible explanation is that the release of EVs in 3D cultures may be partially driven by the higher expression of transporters. The expression of the ATP-binding cassette transporter G1, a cholesterol lipid efflux pump, was reported to be highly expressed in tumoroids of colon adenocarcinoma cells with enhanced stemness. Likewise, the silencing of this transporter blocks the release of EVs and increases the accumulation of intracellular vesicles [61].

Interestingly, cell architecture can be manipulated by applying different collagen concentrations and adding components that are found naturally in the dermis such as fibronectin. Breast cancer cells cultured in 3D following this approach experienced morphological alterations [62]. Moreover, these changes are translated into differences in the cargo of secreted EVs population [62]. Remarkably, there is evidence that 3D culturing presents different gene expression signatures due to the more physiological nanoenvironment of this arrangement. For instance, prostate-derived adenocarcinoma cells (PC-3 and DU145) form large and slowly growing organoids that express multiple stem cell markers, neuroendocrine markers and intercellular adhesion molecules likely to occur in vivo as well. Importantly, 3D cultures promoted the secretion of HSP90 and EpCAM loaded EVs, which are markers of cancer stem cells phenotype [63]. Another example of the physiological environment effect has been observed in cervical cancer 3D cultures. In this case, the EVs were loaded with a small RNA profile comparable (~96% similarity) to in vivo circulating plasma-derived EVs from cervical cancer patients [64].

Moreover, tridimensional architecture allows a better cell orientation and asymmetry. It implies that different populations of EVs loaded with different markers and cargo proteins are released from apical and basal sides of the cells. As an example, organoids derived from colon carcinoma cell line LIM1863 release two types of EVs. Apical EVs are characterized by the presence of EpCAM, and the exclusive identification of the trafficking molecules CD63, mucin 13, and the apical intestinal enzyme sucrase isomaltase, but also an increase in the expression of dipeptidyl peptidase IV and the apically-restricted pentaspan membrane glycoprotein prominin 1 [65]. In contrast, EVs containing the colon epithelial cell-specific A33 marker were enriched with basolateral trafficking molecules such as early endosome antigen 1, the golgi membrane protein ADP-ribosylation factor, and clathrin. These observations are consistent with EpCAM- and A33-EVs being released from the apical and basolateral surfaces of colon carcinoma cells respectively [65].

One of the most important outcomes of the different alterations described here is that cancer cells have a wide range of responses to their environment. In cancer cells response to drugs, a higher release of EVs after chemotherapy treatments has been reported [66]. Interestingly, this observation has been useful to stablish a discovery pipeline of secreted biomarkers in the media of organoid cultures and to identify new protein markers as a response to chemotherapy [66].

Due to the lack of an established biomanufacturing platform for EVs, which is a limitation for clinical translation, one of the most interesting applications of 3D cultures is the large scale and standardizable production of EVs. A simple approach is the use of bioreactor flasks since they increase the production of EVs released by tumoral cells [67]. A more interesting application is to use cell cultures in microfluidic platforms. These automated devices can produce therapeutic exosomes, which could also be engineered, and harvest them in real-time from the on-chip cultures. For instance, this type of tool has been used in leukocytes isolated from human blood [68]. Alternatively, a 3D-printed scaffold-perfusion bioreactor system has been employed to assess the effect of dynamic cultures on the production of EVs from endothelial cells. With this approach the cells were able to maintain their functionality (i.e., pro-vascularization bioactivity or pro-angiogenic gene expression) [69].

## 5. Modelling the Antitumoral Effect of EVs in 3D Cultures

**Figure 2:**
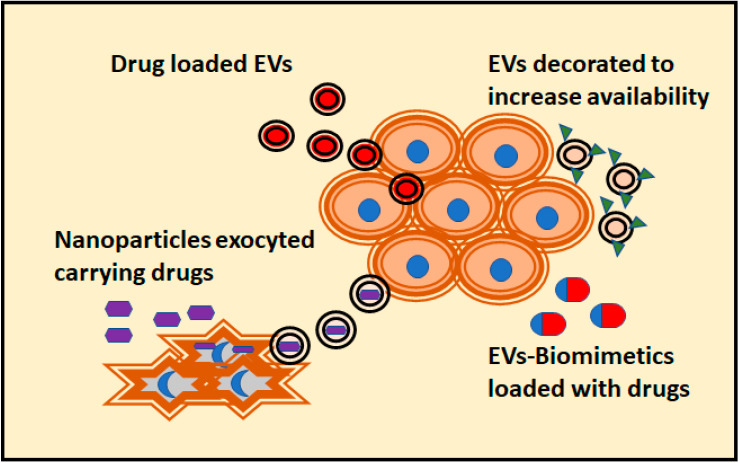
Different strategies attempted to confer antitumoral activity to EVs are depicted. The preferred 3D culture assay for these tests are organoids, with a closer physiological response to in vivo cells. In addition, the penetrance of the drug carried by EVs compared to its free forms can be evaluated. This measurement is not possible to obtain using 2D cultures.

As we mentioned in the previous section, the arrangement of cells in tridimensional conformations often suppose a better physiological model of drug therapy. This is also an advantage to study the application of EVs as potential therapeutical assets against tumoral cells. Up to date, multiple strategies have been designed to increase the antitumoral effect of EVs (Figure 2). According to the literature, most of the 3D culture systems employed to reveal the antitumoral effect of EVs are spheroids or organoids formed with tumor-derived cell lines. Indeed, tumor spheroids are the most common models for testing drug effectivity [70]. Notably, 3D cultures with a single type of cells are likely to exhibit different drug responses than those composed of heterogenous populations of cells [70].

The spontaneous effect of normal cell-derived EVs has been investigated to use them as natural antitumoral agents. For example, glia-derived EVs have shown an antitumoral effect in spheroids of glioma cells by reducing the invasion capacity of the tumor over time [71]. Another example are EVs derived from mesenchymal stroma cells (MSCs) that can inhibit angiogenesis and maintain vascular homeostasis in activated endothelial cells [72]. However, most of the publications focus on the possibility of loading EVs with antitumoral drugs and biomolecules such amino acids, lipoproteins, or nucleic acids. The antitumoral effect of EVs loaded with a specific miRNA (miR-497) has been assayed in a microfluidic device containing a mixture of cells. These types of devices are useful in combination with an extracellular matrix since it allows the study of migration in response to a factor controlled by microfluidic channels [73]. In this case, the cells employed were the non-small cell lung cancer cell line A549 cultured together with human umbilical vein endothelial cells (HUVEC). In these conditions, the tube formation of endothelial cells was inhibited and the migration of the tumor decreased dramatically compared to the control [74]. To avoid limitation of the cocultures associated to cell separation after analysis, both types of cells were separated in the microfluidic devices by Matrigel component. This is a very interesting example of using 3D culturing to mimic the physiological complexity of tumors.

The efforts for loading EVs with antitumoral drugs are well documented in the literature. The importance of EVs as antitumoral agents lays upon the tumor avidity for vesicles. Many approaches consist in loading the EVs with a chemotherapeutic agent. Although still very inefficient, there are different strategies; the simplest methods consist in incubating the drugs with purified EVs [75], and an alternative is to treat parental cells with the drug that would be released by EVs. In a more sophisticated way, a modification on the surface of the EVs allow a targeted loading of the drug (reviewed in [76]). For instance, EVs obtained from endometrial cells have been loaded with atorvastatin and can induce significant apoptotic effects and inhibit the growth of glioblastoma spheroids [77]. Moreover, endothelial cell-derived EVs loaded with meta-tetra(hydroxyphenyl)chlorine can penetrate up to 100 μm in multicellular tumor organoids. Consequently, these EVs increased photodynamic activity, which translates into higher rates of cell mortality [78]. This is a promising result that shows an improvement in the penetration capability compared to liposomes, and hence in the vectorization molecules capabilities. Furthermore, the production of EVs from patient-derived cells is an interesting strategy to overcome many of the problems associated with bioreagent-based therapy. The treatment of melanoma spheroids with macrophage-derived EVs loaded with acridine orange has maintained the delivery of this drug for longer time in comparison to the treatment with free acridine orange [79].

A more sophisticated approach to design antitumoral EVs is their decoration with molecules that promote the interaction between cells and vesicles. This strategy has described an increase of the avidity and specificity of cells to uptake the decorated EVs. For instance, HepG2 cells and human primary liver cancer-derived organoids accumulate more efficiently EVs that have been decorated with tetrahedral DNA nanostructures conjugated with DNA aptamer [80]. These EVs can effectively deliver an engineered cargo, which consists of CRISPR-Cas9 RNA-guided endonucleases, aiming to silence the expression of the protein Wnt-10b. In fact, these EVs inhibited the growth of tumoral cells in vitro [80]. Another strategy was to decorate methotrexate-loaded EVs with Lys-Leu-Ala bound to low-density lipoprotein peptides. The functionalization of the methotrexate-loaded EVs increased the uptake by human primary glioma cell line U87 growing into 3D glioma spheroids and increased the cell mortality rate [81]. Although the use of EVs as carrier of antitumoral molecules is very promising, there are still several limitations. For example, this type of EVs-based drug delivery approach needs accurate isolation methods for those EVs subpopulation that display favorable tropism and the understanding of EVs transport properties is still scarce. In addition, scalable manufacturing remains a major hurdle for clinical translation [82]. A very detailed revision of ongoing clinical trials regarding the use of EVs can be found in [83].

An attempt to overcome some of these problems is the use of EVs mimetics. One of the first solutions to generate “on demand” EVs was to obtain them by shearing cells through a sequential filtering. When loaded with doxorubicin, these EVs mimetics were more effective in targeting ovarian cancer cells in 3D cultures than free doxorubicin. In addition, they showed a higher encapsulation efficiency and drug release over time in comparison to naturally released EVs [84]. Spheroids derived from cancer stem cell have been targeted with tumor-cell-exocytosed nanoparticles made of porous silicon. These synthetic particles loaded with doxorubicin are fed to tumoral cells, which release the nanoparticles with the doxorubicin inside of EVs. This approach greatly improves drug performance over hepatocarcinoma spheroids in comparison to free doxorubicin or the direct use of synthetic particles (Figure 2) [85].

## 6. EVs-Mediated Crosstalk between the Tumor and Cellular Matrix

### 6.1. Tumoral Cells Modify Surroundings Cells through EVs

EVs are released by malignant cells and can further influence the cellular components of the matrix. There are different models employed to study that effect, which vary from low to high complexity. Synthetic gel cultures are the most employed models to study the invasion or tubule formation in cultures with a single type of cell models. A classic example of these type of models is represented by the treatment of HUVEC cells cultured on Matrigel with EVs derived from chronic myelogenous leukemia. It causes the reorganization of HUVEC cells into tubes [86] but also, the movement of EVs within and between nanotubular structures further connecting the remodeled endothelial cells [87]. In addition, the effect of renal cancer cell-derived EVs, which induce VEGF expression in HUVEC cells has also been described [88].

Regarding the tumor-matrix crosstalk, MSCs probably are the most interesting players due to their response to EVs treatment in bioengineered 3D microenvironments. EVs derived from MDA-MB-231 (metastatic breast cancer cell line) have shown to convert MSCs into tumor-activated MSCs. This results in an immunomodulatory phenotype that was particularly prominent in response to bone-tropic cancer cells. In contrast, MCF7 (considered a non-metastatic breast cancer cell line) -derived EVs failed to generate this phenotype in the MSCs culture [89]. It has also been reported that colorectal EVs induce alterations in colonic MSCs morphology and increase MSCs proliferation, migration, and invasion. Colorectal EVs also provoke a higher ability to form spheroids, and an impact on the metabolic respiration by the acidification of the extracellular environment associated with a plasma membrane redistribution of vacuolar H^+^-ATPase. They also increase the expression of the carcinoembryonic antigen. These modifications suggest that colorectal cell-derived EVs are able to activate MSCs to favor tumor growth and malignant progression [90]. Likewise, the treatment of ovarian cancer spheroids with cisplatin showed a release of EVs that can alter MSC cells. These MSCs displayed an increase in the migration pattern and secreted more amount of interleukin-6 (IL-6), interleukin-8 (IL-8), and VEGFA. Moreover, bone marrow MSCs induce angiogenesis in endothelial cells and the migration of low-invasive ovarian cancer cells upon contact with EVs [91].

**Figure 3:**
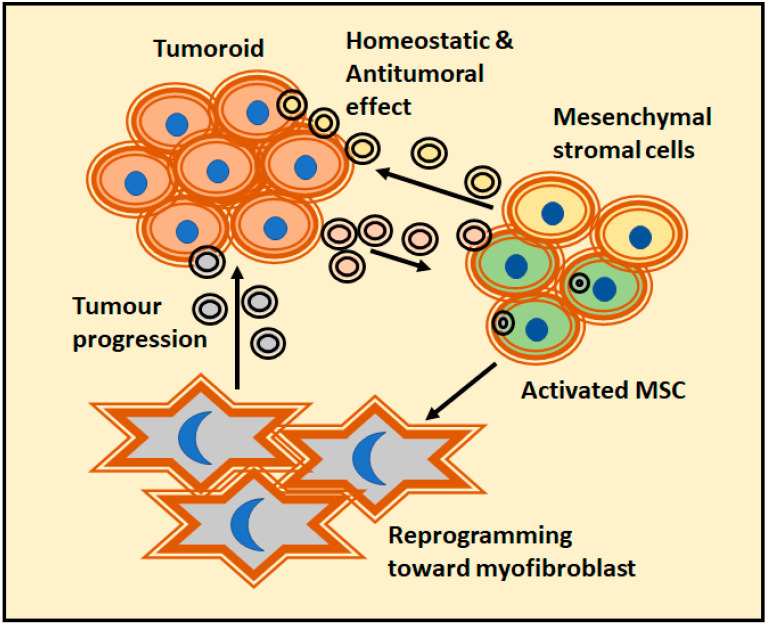
A clear example of tumor-matrix interaction was provided by the prostate cancer models and their action over mesenchymal stroma cells (MSCs). It is a complex relationship were EVs released by tumors could change the fate of MSC. While MSC-derived EVs have a homeostatic effect, activated MSCs usually reprogram themselves into myofibroblast and actively favor tumor progression.

The effect of prostate cancer EVs dominates a new program of MSC differentiation that impairs both the classical adipogenic differentiation and the skewing differentiation towards alpha-smooth muscle actin (αSMA) positive myofibroblastic cells (Figure 3). The differentiated MSC performed pro-angiogenic functions and enhanced the tumor proliferation and invasivity in a 3D co-culture model. In this case, the differentiation was dependent on TGFβ containing EVs. Remarkably, a comparable dose of soluble TGFβ could not generate the same phenotype [92]. Tumor-derived EVs can target fibroblasts directly. Moreover, early-stage primary colorectal adenocarcinoma cells have shown to be unable to invade Matrigel matrix themselves. Alternatively, they secrete EVs to reprogram normal fibroblasts and acquire a de novo capacity to invade first the matrix and thence, the adenocarcinoma cells. It is worth to mention that EVs upregulate fibroblast proteins implicated in focal adhesion, regulators of actin cytoskeleton and signaling pathways important in pro-invasive remodeling of extracellular matrix [93]. In addition, epithelial cells can be transformed by tumoral EVs; a recent study described how human peritoneal mesothelial cells treated with epithelial ovary cancer-derived EVs accumulate miR-99a-5p. The presence of miR-99a-5p drives the invasion in a 3D Matrigel culture model due to a higher expression levels of fibronectin and vitronectin [94].

Multiple cellular models are more complex than single cell cultures and are interesting structures to mimic tumor complexity. The development of multicellular lung organoids mimicking the lung microenvironment with air sac-like structures and lung surfactant proteins has shown that pretreating the cells with tumor exosomes triggered cancer cell colonization. Notably, the sensitivity to drug therapy described in this multicellular model is closer to in vivo observations rather than the sensitivity obtained with 2D or single cell 3D cultures [70]. The other major advantage of multicellular cultures is that they allow the study of different interactions simultaneously. However, it is worth to remark that these desirable characteristics can only be achieved through an extensive experimental testing, and such characterization is challenging, since synthetic multi-population systems are among the most complex systems described to date [95]. A successful example is the design of a microfluidic device with multiple cells that mimics the tumor microenvironment in situ, including extracellular matrix (ECM), interstitial flow and environmental EVs. Such device has been employed to study the endothelial-mesenchymal transition (EMT). The number of cancer-associated fibroblasts (CAFs) differentiated from HUVECs increased upon treatment with melanoma-derived EVs, hence promoting EMT. The negative pro-tumorogenic effect of cancer-derived EVs over HUVEC cells could be intensified by the enrichment of miR-221-3p containing EVs [96]. In cervical squamous carcinoma cells derived EVs, vesicular miR-221-3p promoted angiogenesis in Matrigel tube formation assay, but also an increase in spheroid sprouting and migration, and induced a faster wound healing. Moreover, cancer stem carcinoma cell-derived EVs transport miR-221-3p from cancer cells to vessel endothelial cells and promote angiogenesis by downregulating the protein THBS2 [96]. On another hand, the transformation from fibroblast to myofibroblast can be impaired by depleting RAB35. The phenotype of the remaining EVs population is insufficient to drive the fibroblast to myofibroblast differentiation, showing attenuated motile behaviors in 3D in vitro models [97]. In contrast, MSCs derived EVs suppress EMT, maintain vascular homeostasis, and ultimately lead to the recovery of CAFs back to endothelial cells [72].

### 6.2. Tumoral EVs Modify the Tumoral Cells

Thanks to the efficient way of capturing EVs, tumor cells have the ability to acquire or recover phenotypes from other cancer cells by recycling the cargo contained in their EVs. In this respect, MMP3 is an interesting molecule. MMP3 is a matrix metalloproteinase that enhances proliferation and tumorigenesis. Lung metastatic tumoroid cells with MMP3 knocked out (MMP3-KO) showed a significant reduction in tumoroid size and they developed a necrotic area within tumoroids. However, when MMP3-KO cells were treated with EVs from the original lung metastatic line, they recovered expression of MMP3, but also CD9 (a vesicular marker) and Ki-67 (proliferation marker) [98]. In another study, hypoxia have shown to enhance the release of EVs from colorectal cancer cells in an Hypoxia Induced Factor (HIF1)-dependent manner, and these EVs further stimulated motility, invasiveness and stemness of primary tumor cells SW480 [99]. A study with pancreatic ductal adenocarcinoma cells (PDAC) have demonstrated that the presence of asparaginyl endopeptidase-containing EVs derived from adenocarcinoma enhanced the invasive ability of PDAC cells, whereas EVs lacking that molecule decreased their invasive ability [100]. In the case of gastric cancer cells, the release of EVs with either high or low CD97 has been described. These two types of EVs had differences on their promotion of tumor invasion in Matrigel cultures in a dose-dependent manner. This supports the protumoral effect of CD97 in this model [101].

Some melanoma diseases overexpress RAB27A, a well-known protein in the mechanism of the formation of exosomes through the endocytic pathway. The silencing of RAB27A in melanoma cell lines caused an inhibition of 3D spheroid invasion and cell motility in vitro, as well as the spontaneous metastases in vivo. Interestingly, the effect can be reverted by using RAB27A-replete EVs. However, the effect cannot be reverted if EVs from melanoma RAB27A knockdown cells are used instead, suggesting that this gene is the responsible of promoting a population of pro-invasive EVs [102]. A similar interplay phenomenon can be observed using claudin-loaded EVs in claudin knockdown cells [103]. Remarkably, there is more evidence that tumoral cells are influenced by tumor-derived EVs. A 3D co-culture performed with original colorectal adenocarcinoma cell line and cellular subclones resistant to 5-fluorouracil demonstrated naïve spheroids release EVs loaded with miR-200 family members. This miRNA family is well-known to repress EMT and consequently, attenuate plasticity and migration. Horizontal miR-200 signaling prevented resistant adenocarcinoma tumor spheroids to disrupt the continuous lymphendothelial cell layer. In addition, they lost the ability to generate a circular chemorepellent-induced defect [104]. On contrary, EVs from 5-fluorouracil resistant colorectal carcinoma, which are devoid of miR200, accelerated circular chemorepellent-induced defects [105].

### 6.3. Effect of EVs Released by the Cellular Component of the Matrix over Tumor Cells

A common strategy to study the effect of EVs released by the cellular component of the matrix is to generate organoids or spheroids using tumor cells. This could be also translated into clinical approaches; for instance, the generation of organoids with patient-derived colorectal cancer cells organoids. Fibroblast-derived EVs induce colony formation of colorectal carcinoma organoids under hypoxia. In contrast, there is no major effect of tumor-derived EVs on the activation of fibroblasts [60]. Fibroblast-derived EVs induce cell proliferation (in an epidermal growth factor-dependent manner) to colorectal cancer patient-derived organoids, and the data pointed to vesicular amphiregulin as a major factor in inducing cell proliferation [106]. The EVs derived from macrophages had also an effect over tumoral cells, and the effect increases when the release of EVs is induced by deoxycholic acid treatment. EVs released in such condition increases the expression of spasmolytic polypeptide-expressing metaplasia markers (TFF2 and GSII lectin) in gastric organoids compared to EVs derived from macrophages without deoxycholic acid stimulation [107].

It has been also described that EVs secreted by MSCs obtained from patients with oral leukoplakia and dysplasia, or oral carcinoma, exhibited induction of proliferation, migration and invasion of oral carcinoma cells in 3D coculture. This effect is significantly higher than the one obtained co-culturing carcinoma cells and normal oral mucosa MSCs [108]. The homeostatic and antitumoral role of healthy MSC-derived EVs has been documented in complex 3D cultures ultimately reverting CAFs back to endothelial cells [72]. However, it has been also described that MSCs were capable to stimulate human glioblastoma cell proliferation through a paracrine effect mediated by TGFB1. Moreover, MSCs in direct cell-cell contact with glioblastoma cells provoked an increased proliferative and invasive tumor cell behavior under 3D culture conditions [109].

## 7. Conclusions

In this review, we have described examples in which different 3D culture strategies are employed to assess the effect of EVs over cells. The most common strategies are gel-based cultures; both spheroid and organoids are used depending on the cell complexity. However, there are also examples of microfluidic systems and bioreactors. These experiments showed that cells in a 3D culture system behave differently than in 2D cultures, often in a more similar manner to in vivo conditions. This should be taken in account when performing studies relative to drug sensitivity or EVs release. The possibility of implementing co-cultures in 3D models allows complex interactions and obtaining results relative to the cellular crosstalk, but we should also remark that such models require extensive characterization. By using co-cultures, it can be described how tumoral EVs can modify cells from the matrix to display antitumoral activity, but also induce the release of EVs that feeds back the activation of tumoral cells itself. In addition to the description of tumor biology, other important application of 3D models focusses on the study of EVs as chemotherapy carriers. The use of organoids and spheroids as models allows the measure of drug penetrance, and the observed cell sensitivity to those drugs seems to be closer to in vivo results, when compared to studies using cell monolayers. Another point where 3D cultures can help is to solve the problems inherent to scale the production. Notably, bioreactors are of increasing interest as a source of standardized and scalable production platforms of EVs. Although there is still a long way to solve all the technical challenges, the adoption of 3D culture models will bring a qualitative improvement on the discovery of potential applications of EVs in cancer research.

## Figures and Tables

**Table 1:** Main advantages and limitations of the different cellular models in cancer research [13,14].

Model	Advantages	Limitations
2D Monolayers	Easy and cost effective Large amount of data available Reproducible cultures, easy to work for downstream applications and imaging	Reduced cell-to-cell interactions Different sensitivity to drugs Loss of biological characteristics over time
Gel based 3D Cultures	Cell–ECM interactions Possible to incorporate different factors in the gel, extending release over time Uniform spheroids/organoids	Difficult to dispense cells Change of growth media could be irregular Difficult to retrieve cells and downstream analysis
Low-attachment plates	Simpler and cheaper when compared to gel based systems Long-term culture	Time consuming and low yield achieved Heterogenous spheroids
Microfluidic systems	Possible chemical gradients Control of fluid rates Convenient for multicellular cultures controlling cell locations	Expensive commercial devices or not well-characterized “in house” build devices Fluidic problems related to bubbles and clogging

## Data Availability

Not applicable.
